# Media coverage of Robin Williams’ suicide in the United States: A contributor to contagion?

**DOI:** 10.1371/journal.pone.0216543

**Published:** 2019-05-09

**Authors:** Victoria Carmichael, Rob Whitley

**Affiliations:** Department of Psychiatry, Douglas Mental Health University Institute, McGill University, Montreal, Quebec, Canada; Stellenbosch University, SOUTH AFRICA

## Abstract

Evidence suggests that suicide rates can increase following the suicide of a prominent celebrity or peer, sometimes known as ‘suicide contagion’. The risk of contagion is especially high when media coverage is detailed and sensational. A recent study reported a 10% increase in U.S. suicides in the months following the suicide of comedian Robin Williams, who died in August 2014. The authors tentatively linked this increase to sensational media coverage; however, no content analysis of U.S. media was performed. As such, the aim of the present study is to formally examine the tone and content of U.S. newspaper coverage of Williams’ suicide. The primary objective is to assess adherence to suicide reporting guidelines in U.S. newspapers after his suicide. The secondary objective is to identify common emerging themes discussed in these articles. The tertiary objective is to compare patterns of results in the U.S media with those in the Canadian media. Articles about Williams’ suicide were collected from 10 U.S. newspapers in the 30-day period following his death using systematic retrieval software, which were then examined for adherence to suicide reporting recommendations. An inductive thematic analysis was also undertaken. A total of 63 articles were included in the study. We found that 100% of articles did not call it a ‘successful’ suicide, 96.8% did not use pejorative phrases and 71% did not say ‘commit’ suicide. However, only 11% included information about help-seeking, 27% tended to romanticize his suicide and 46% went into detail about the method. The most prominent emerging theme was Williams’ struggles with mental illness and addiction. These findings suggest that U.S. newspapers moderately adhered to best practice recommendations when reporting Williams’ suicide. Key recommendations were underapplied, which may have contributed to suicide contagion. New interventions targeting U.S. journalists and media may be needed to improve suicide reporting.

## Introduction

Suicide remains a serious public health issue and is a leading cause of death across the world [1,2]. Some research indicates that suicide rates significantly increase following the suicide of a prominent celebrity or peer: a phenomenon known as ‘suicide contagion’, ‘copy-cat suicides’ or the ‘Werther-effect’ [3,4].

Evidence suggests that suicide contagion may be particularly pronounced when media coverage of a peer/celebrity suicide is detailed and uses sensational language [5,6,7]. These studies theorize that some at-risk consumers of such media coverage identify with the celebrity/ peer and their struggles. This is especially so when the consumer perceives demographic similarity and characterological homophily with the celebrity/peer [8]. Such affinity, combined with sensational and detailed media coverage, may lead some at-risk consumers to conclude that suicide is an acceptable solution to ongoing struggles, or even a heroic and decisive act worthy of emulation. It is posited that this can increase suicidal behaviour in such at-risk individuals [4,6,9].

Interestingly, this theory is supported by several studies indicating that people who die by suicide after a celebrity/ peer suicide are significantly more likely to use the same suicide methods (and sometimes the same location), the details of which may have been learnt through sensational and detailed media coverage [7,10,11]. This ‘copy-cat’ behaviour suggests some level of affinity with the celebrity/ peer and their actions.

These issues recently made international news thanks to a well-publicized and carefully-crafted epidemiological study examining suicide contagion in the United States (U.S.) following Robin Williams’ suicide [12]. Robin Williams was a much-loved comedian and actor who died by suicide (suffocation through hanging) on August 12 2014. This study examined the monthly suicide count before and after this suicide, finding a 10% increase in suicides in the two months following his death. A significant increase in the number of suicides by suffocation in men was also observed. The authors speculated that this increase could be partially attributed to detailed and glamourized U.S. media coverage of Williams’ suicide provoking copy-cat suicides, however the authors did not conduct a content analysis to examine media coverage.

In contrast, researchers have systematically examined the tone and content of Canadian media coverage of Robin Williams’ suicide in a focused study [13]. In this study, researchers assessed newspaper article fidelity to evidence-based recommendations for reporting suicide (see Table 1). These recommendations were produced by the Canadian Journalism Forum on Violence and Trauma, in collaboration with the Canadian Broadcasting Corporation and the Mental Health Commission of Canada, published in a booklet called ‘Mindset’ [14].

**Table 1:** *Mindset* recommendations.

Recommendations
Do consider whether this particular death is newsworthy.
Do look for links to broader social issues.
Do respect the privacy and grief of family or other survivors.
Do include reference to their suffering.
Do tell others considering suicide how they can get help.
Do not shy away from writing about suicide.
Do not romanticize the act.
Do not jump to conclusions.
Do not suggest nothing can be done.
Do not go into details about the method used.
Do use plain words.
Do not say the person committed suicide.
Do not call suicide successful or attempted suicide unsuccessful.
Do not use or repeat pejorative phrases.

Although produced in Canada, the *Mindset* recommendations overlap with other prominent suicide reporting guidelines, such as those produced by the World Health Organization (WHO) [15] and the U.S. based ‘Recommendations for Suicide Reporting’ [16]–a set of recommendations co-created by numerous stakeholders including U.S. government agencies, universities and non-governmental organizations that are publicly available on a dedicated website. Common recommendations across these guidelines include ‘avoid language that sensationalizes or romanticizes the suicide’, ‘provide information about seeking help’ and ‘avoid using terms such as ‘commit suicide’ or ‘successful suicide”.

Interestingly, the Canadian research found that journalists strongly adhered to *Mindset* guidelines when reporting Robin Williams’ suicide [13]. In fact, study results showed that 85% of the newspaper articles included in the study applied at least 70% of the recommendations when covering Robin Williams’ suicide. Notably, 76% of articles did not go into details about the suicide method used and 86% did not use language that romanticized or glamourized the act.

These findings overlap with results from studies in other western jurisdictions. For example, a recent study assessed British media coverage of suicide, finding that 69% of articles did not go into detail about the method used, and that 83% avoided ‘sensationalist or irresponsible language’ [17]. Similar results were found for a study on the Irish media, finding that 80% did not go into detail about the method used, and 88% avoided sensationalized language [18].

In contrast, studies from non-western jurisdictions indicate more frequent violations of suicide reporting guidelines. In South Korea, a study indicated that only 32% did not go into detail about the method used, though 76% avoided ‘sensational coverage’ [19]. Likewise, a study of the Pakistani media found that 66% of the articles did not go into details about the method used, and 99% of the articles used ‘careless language’ [20].

To our knowledge, there has not been any recent systematic analysis of U.S. media coverage of suicide and adherence to commonly-used suicide reporting guidelines. This is concerning as over 47 000 Americans died by suicide in 2017, and U.S. suicide rates have risen steadily since 1999 [21,22]. Moreover, Fink et al [12] speculate that U.S. media coverage of Robin Williams’ suicide may have been overly sensational and detailed, given the observed increase of suicides after Robin Williams’ death; however, this has not yet been studied systematically. Indeed, it is unknown whether the U.S. media is reporting suicide in a similar way to other comparable countries such as Canada. This is important to know given the national and international influence of the U.S. media and its potential contribution to suicide contagion.

All this raises an important research question: how did the U.S. media report the suicide of Robin Williams? This research question propels the current study, which aims to formally examine the tone and content of U.S. newspaper coverage of Robin Williams’ suicide through deductive and inductive analysis. The primary objective is to assess adherence to suicide reporting guidelines in U.S. newspapers after Robin Williams’ suicide through deductive assessment. The secondary objective is to identify common themes inductively emerging in these newspaper articles which may not be captured by the *Mindset* guidelines. The tertiary objective is to compare patterns of coverage of Robin Williams’ suicide in the U.S. media as compared to the Canadian media.

## Methods and materials

The data for this study consisted of U.S. newspaper articles reporting Robin Williams’ suicide. These articles were obtained using FP Infomart, a constantly updated and comprehensive media monitoring software which allows users to search and retrieve full-text newspaper articles using key words [23]. Articles mentioning the terms ‘suicide’ and ‘Robin Williams’ were collected from 10 U.S. print newspapers in the 30-day period following his death (12 August 2014 to 10 September 2014). This was the same date range and similar search terms to the aforementioned Canadian study. Newspapers were purposely chosen to include nationally-read publications such as USA Today, the New York Times and the Washington Post; as well as prominent regional publications in major cities such as the Boston Globe and the Miami Herald.

A sample of ten newspapers is an optimal number as it gives breadth and variety, while ensuring that data is manageable and analysis is focused. Such an approach can provide a broad overview of the media/ suicide landscape, and ten is a similar number of newspapers to other studies analyzing media coverage of suicide and mental health [20,24]. The ten newspapers are listed in S1 Appendix. Articles were excluded from analysis if they only made a passing or incidental reference to his suicide, and if they were exact duplicates of earlier versions.

Each article was then assessed for adherence (yes) or non-adherence (no) to the *Mindset* recommendations (see Table 1). Two recommendations were excluded from the analysis as they are pre-publication rather than content recommendations, namely “Do consider whether this particular death is newsworthy” and “Do not shy away from writing about suicide. The more taboo, the more the myth”. After coding the articles, frequency counts and percentages were calculated for adherence/ non-adherence to each of the 12 *Mindset* recommendations.

All articles were read and coded by the first author, an experienced analyst with years of experience coding newspaper articles and leading studies related to media coverage of mental health and suicide [25,26]. This author received intense training and close supervision from the second author for the present study. Firstly, the first author read seminal papers about suicide reporting in the media and familiarized herself with *Mindset*. Secondly, the second author created a formalized codebook with examples to operationalize the *Mindset* guidelines, which were used in informal training exercises with the first author (see S1 File). Thirdly, a sub-sample of news stories (20%; N = 13) was double-coded by both authors using this codebook to assess interrater reliability. Cohen’s kappa for agreement between the two raters was 0.76, which suggests substantial agreement [27]. This high degree of overlap was unsurprising, given that the recommendations are mainly simple injunctions, e.g. ‘do not say the person committed suicide’ and ‘do not call suicide successful or attempted suicide unsuccessful’.

Although the *Mindset* recommendations were produced for the Canadian media, we used them in the present study for three reasons. First, usage of *Mindset* allows us to directly compare U.S. results with those observed in the aforementioned Canadian study, in line with objective three of the study [13]. Second, the *Mindset* guidelines include core recommendations that were used in other similar research mentioned in the introduction, including those in the U.K, Ireland, Pakistan and South Korea. This means that further comparisons can be meaningfully drawn between results from the present study and results from other jurisdictions. Third, the *Mindset* guidelines overlap with the previously mentioned U.S. based ‘Recommendations for Suicide Reporting’ [16]; for example, both sets of guidelines include recommendations to avoid sensationalistic language, to include information about help-seeking resources, and to eschew referring to a completed suicide as a ‘successful’ suicide.

Finally, we conducted an inductive content analysis to identify and label major themes across the dataset, following standard procedure in qualitative analysis [28,29,30]. This was done in order to capture any themes emerging from the data that may not have been covered through assessment of the 12 *Mindset* guidelines, in line with the overall aim of the study, which is to examine the tone and content of U.S. newspaper coverage of Robin Williams’ suicide. Adding such an inductive sub-analysis to a deductively-driven study is a recommended practice, as it overcomes the conceptual rigidity associated with using a limited number of pre-defined codes to assess content [31]. Indeed, such an approach was used in the aforementioned Canadian study, and the present study followed similar analytical procedures, again offering a useful point of comparison between the U.S. and Canadian data. Moreover, any inductively emerging themes can point to areas of discussion that can feed into the revision of suicide reporting guidelines.

For this inductive analysis, the first author carefully read each article, systematically noting key content about Williams’ suicide that regularly appeared in different articles, especially focusing on emerging inductive content that was not adequately captured by the 12 *Mindset* recommendations. Examples of such emerging content includes inferred causative factors in his suicide or specific narratives related to the chain of events leading up to his death. This involved the creation of codes that summarized such key content through ‘open coding’, leading to a list of codes that provisionally described emerging patterns across the data-set. These codes were verified by the second author who again read a sub-set of articles to corroborate the observations made. Both authors then discussed these codes, collapsing them into a shorter list of cross-cutting and over-arching themes. The first author then engaged in a second round of supervised ‘focused coding’, which involved rereading the articles to formally enumerate the presence or absence of the identified themes in each separate article. These themes were then ranked in order of prominence. This process of intensive training and close supervision, multiple coding and systematic step-by-step analysis follows recommended procedures for maintaining rigour in inductively-driven qualitative research [30,32].

## Results

A total of 93 articles were retrieved using the raw search terms. Thirty articles were rejected after applying the exclusion criteria detailed above. This led to a total of 63 articles being included in the analysis (listed in S2 Appendix).

Overall frequency counts and proportions are given in Table 2 alongside figures from the previously described Canadian study examining adherence to *Mindset* guidelines for Robin Williams’ suicide in the Canadian media. As can be seen, articles tended to follow many of the recommendations. For example, 100% of the articles did not call it a ‘successful’ suicide, 96.8% did not use pejorative phrases, and 71% did not say ‘commit’ suicide. That said, substantial proportions of the articles violated many of the recommendations. For example, only 11% gave information about where to seek help, and 27% tended to romanticize the act of suicide such as describing memorials outside his home, glamorously linking comedy to suicide or implying that the suicide was the heroic act of a misunderstood and sensitive artist. Perhaps most importantly, almost half of the articles (46%) described the method used, a practice strongly discouraged in *Mindset* and other recommendations. As can be seen in Table 2, U.S. media had lower proportions of ‘yes’ responses on key variables in comparison with Canadian media, such as ‘do not go into detail about the methods used’ and ‘do not romanticize the act’.

**Table 2:** Levels of adherence to *Mindset* guidelines in the U.S. and Canada in articles discussing Robin Williams’ suicide.

	United States (N = 63)	Canada (N = 66)
Recommendation	Yes (%)	Yes (%)
Do consider whether this particular death is newsworthy.	n/a	n/a
Do look for links to broader social issues.	39 (61.9)	52 (78.7)
Do respect the privacy and grief of family or other survivors.	44 (69.8)	63 (95.5)
Do include reference to their suffering.	33 (52.4)	63 (95.5)
Do tell others considering suicide how they can get help.	7 (11.1)	18 (27.3)
Do not shy away from writing about suicide.	n/a	n/a
Do not romanticize the act.	46 (73.0)	57 (86.4)
Do not jump to conclusions.	40 (63.5)	43 (65.2)
Do not suggest nothing can be done.	56 (88.9)	60 (90.9)
Do not go into details about the method used.	34 (54.0)	50 (75.8)
Do use plain words.	50 (79.4)	58 (87.9)
Do not say the person committed suicide.	45 (71.4)	51 (77.2)
Do not call suicide successful or attempted suicide unsuccessful.	63 (100.0)	61 (92.4)
Do not use or repeat pejorative phrases.	61 (96.8)	65 (98.5)

Cumulative percentages of U.S. articles meeting each recommendation are given in Table 3. Of the 63 articles analyzed, all of them followed at least five of the recommendations. Just over 70% of the articles followed at least 8 of the recommendations, and 43% of the articles followed 9 or more of the recommendations. Only 22% of the articles followed 10 or more of the recommendations. Notably, none of the articles applied all 12 recommendations.

**Table 3:** Cumulative proportion of articles meeting each recommendation.

Guidelines met	Number of articles	Percent of articles (N = 63)
All 12	0	0.0
11+	3	4.8
10+	14	22.2
9+	27	42.9
8+	45	71.4
7+	54	85.7
6+	60	95.2
5+	63	100.0
4+	63	100.0
3+	63	100.0
2+	63	100.0
1+	63	100.0

### Qualitative analysis

The inductive analysis resulted in five themes, which are listed in Table 4 in order of prominence, along with frequency counts and percentages. A total of 47 articles (75%) discussed Williams’ struggles with mental illness and addiction including depression, alcohol and substance abuse. Common narratives included “Williams’ closest friends and colleagues knew well that beneath his manic, Technicolor exterior, the actor had battled depression for years…and fought to maintain a sobriety that had at times proved fragile…” (A30). A similar example is “While Robin Williams was open about his battle with depression, none of us knew how bad it was until the comic genius, who struggled for many years with his illness and substance addiction, committed suicide this week” (A10). These articles often referred to Williams’ mental health struggles in a nuanced and poignant manner, consistent with the *Mindset* guideline ‘do include reference to their suffering’ but specific to the mental suffering associated with a chronic mental illness. That said, some of these articles simplistically concluded that mental illness was the sole cause of the suicide, and these were coded ‘yes’ to the *Mindset* guideline ‘do not jump to conclusions’.

**Table 4:** Themes emerging from the inductive analysis.

Theme	Number of articles	Percent of articles (N = 63)
*Past struggle with mental illness and addiction*	47	74.6
*Respectful remembrance of Robin Williams life and work*	44	69.8
*The dark side of comedy*	22	34.9
*Explicitly and highly-detailed description of suicide method*	16	25.4
*Need to start conversation about suicide/mental health*	15	23.8

Further, 70% of articles respectfully remembered the life and work of Williams as a beloved comedian and actor. Some reminisced on specific roles in film and television, writing how “his characters made me laugh, cry and remember to keep life in perspective” and “gave the world countless moments of joy for almost four decades” (A5). Likewise, several articles hailed Williams as a comic “genius” and “master” (e.g., A7, A10, A15, A47). Many included tributes by fellow comedians, actors or statements from the public. Others memorialized his life by recounting details of his childhood, professional career, marital life as well as various physical health issues including an open-heart surgery (A11, A29, A48, A54). This theme of ‘respectful remembrance’ was not adequately captured by any single *Mindset* guideline.

The dark side of comedy also emerged as a prominent theme, appearing in 22 articles (35%). Many discussed how comedians often suffer from mental health issues and that comedy can be used to hide, or ‘mask’ these struggles. Common narratives included “Comic mania can mask darkness with joy” (A29) and that “Williams was a showman—perhaps the most vulnerable of public persons …”, describing his demeanor after comedic performances as “deflated, exhausted and spent…smaller than his screen self” (A15). This theme is not adequately captured by any *Mindset* guideline, perhaps due to the rarity of comedian suicides.

As mentioned, nearly half of the articles violated the injunction ‘do not go into details about the method used’. However, the inductive analysis revealed that this injunction was sometimes violated in a highly-explicit and descriptive manner. We classified these instances of highly-explicit description as a theme in its own right, given its link to suicide contagion in previous studies [5,6,7]. Indeed, one in four (25%) described the suicide method in an explicitly highly-descriptive manner; informing readers about exactly how and where he died in intense detail. For example, one article described how “[Williams] was found dead in his bedroom, clothed, slightly suspended in a seated position with a leather belt around his neck, with one end wedged between a closet door and door frame” (A33) and another wrote that “…we struggle with the notion that someone so talented, so rich and so universally beloved would be so desperate to die that he’d try to slit his wrists with a pocket-knife and hang himself in a closet when that failed” (A11).

Lastly, a total of 15 articles (24%) recognized the need for open dialogue and discussion about suicide and mental health. One article, for instance, wrote that “the best way to honor Williams may be to drag depression out of the closet and place it center stage” (A11) and another how “mental health advocates say they hope [Williams’ death] will also renew efforts to prevent suicide” and “spur discussion” (A37). This theme included articles that used Robin Williams’ suicide as an opportunity to raise awareness of suicide prevention and reduce mental health stigma. Again, this theme was not adequately captured by any single *Mindset* guideline.

## Discussion

A key finding of this study is that U.S. newspaper articles about Robin Williams’ death have only moderate levels of adherence to core suicide reporting recommendations. On the one hand, all articles applied at least 5 of the 12 *Mindset* recommendations. On the other hand, over half of the articles failed to apply at least 9 recommendations.

Perhaps most importantly, almost half (46%) ignored the recommendation ‘do not go into detail about the method used’, with one-quarter of the articles describing the suicide in an explicit and detailed manner, so much so that ‘highly-detailed description’ emerged as a theme in its own right in the qualitative analysis. This recommendation to avoid detail about the method used is not only found in *Mindset*, but also in other guidelines such as the internationally-applicable WHO guidelines and the British Samaritans Media Guidelines for Reporting Suicide [15,33]. Indeed, this recommendation is considered essential for suicide prevention as several studies indicate a link between detailed reporting on a celebrity suicide method and subsequent suicide in the community by such method [5,34,35].

Of note, the 46% of U.S. articles violating this recommendation is considerably higher than that seen in similar media research from other jurisdictions mentioned in the introduction. For example, the media in Ireland (20%), Canada (24%), the U.K. (31%) and Pakistan (34%) had a much lower frequency of violations of this recommendation when reporting suicide per se [13,17,18,20].

Our other key finding was that several prominent themes emerged across the articles which were not adequately captured by the *Mindset* guidelines. This included Williams’ struggles with mental illness and addiction, respectful remembrance of his life and work, as well as the need for open dialogue and discussion about suicide and mental health. All three of these themes could be considered helpful from a public health perspective. Adopting such angles has been identified in previous research as an important means of raising awareness of suicide prevention and reducing stigma associated with mental illness in the news [13,25].

Hence, using the tragedy of an individual suicide to open dialogue and discussion about suicide prevention and mental health in a news article could be a candidate recommendation in any future revision of *Mindset* and other suicide-reporting guidelines [25]. Likewise, utilizing ‘respectful remembrance’ in an article humanizes the deceased individual in a poignant and reflective manner and should be encouraged as part of responsible reporting.

An advantage of our methodology is that it allows direct comparison with the results from an analogous study examining adherence to *Mindset* recommendations in Canadian newspaper articles about Robin Williams’ suicide [13]. Overall, this comparison indicates that Canadian articles had much higher rates of adherence in comparison to the U.S. articles. For example, almost double the number of U.S. articles went into detail about the method used: 46% of U.S. articles vs 24% of Canadian articles. Similarly, almost double the amount of U.S. articles (27%) romanticized the act, compared to 14% of the Canadian articles. Also, 27% of Canadian articles gave information about where and how to seek help, almost three times higher than U.S. articles (11%). In total, 85% of Canadian newspaper articles applied 70% or more of the 12 recommendations, almost double the figure (43%) seen in the U.S. articles.

Likewise, the methodology allows for a comparison of qualitative themes emerging from the Canadian and U.S. articles. Again, differences were observed. In Canada, an emerging theme was ‘the need to open the conversation’, observed in 45% of articles. This is similar to the theme in the present study, ‘need to start conversation about suicide/ mental health’ which was observed in only 24% of U.S. articles. That said, the U.S. and Canadian newspapers shared some similar prominent themes, such as discussion of addictions and the dark side of comedy.

This pattern of reporting may have contributed to the increased suicide rates observed by Fink et al in the months following Robin Williams’ suicide [12]. As mentioned, detailed and romanticized description of celebrity suicides has been liked to suicide contagion in previous studies [5,7,34,35]. The fact that Williams was a beloved national figure, combined with this mixed pattern of reporting, may have negatively affected vulnerable readers who identified with Robin Williams and his struggles, especially in readers perceiving demographic similarity and characterological homophily [8]. Such vulnerable readers may have concluded from newspaper articles that suicide is an acceptable (or even heroic) solution to ongoing struggles. This could have contributed to the observed increase in suicide, however such links are speculative and further real-time research is necessary to explore the relationship between celebrity suicide, associated media coverage and subsequent community suicides.

These findings have numerous public health implications. First, they suggest the need to revise existing suicide reporting guidelines. Notably, there have been some changes to U.S. suicide reporting guidelines since Williams’ death. The widely-used Associated Press Stylebook now recommends “suicide stories, when written should not go into detail on methods used” [36]. Similar changes could be made to the on-line ‘Recommendations for Reporting on Suicide’ mentioned in the introduction. The guideline ‘do not go into detail about the method used’ is not explicitly stated in these recommendations, but implicitly alluded to across each guideline. For instance, these guidelines suggest that journalists write “Kurt Cobain dead at 27” instead of “Kurt Cobain used shotgun to commit suicide”, without reference to the higher-order principle ‘do not go into details about methods used’ [16]. In light of our findings and the previously described research on contagion, these recommendations could be re-written to include a specific bullet point ‘do not go into details about the method used’.

Similarly, the qualitative analysis of both the U.S. and Canadian data indicates that many journalists used the tragedy of Robin Williams suicide to open helpful dialogue and discussion about suicide prevention, mental health stigma and wider issues of health care. From a public health perspective, such efforts are welcome as they can raise awareness and catalyze change [25,37]. As such, future revisions of guidelines may include a new bullet point along the lines of ‘do discuss suicide prevention, mental health and health care where appropriate.’ Such a positively-worded ‘do’ recommendation encourages creative freedom among journalists, and may be especially important in guidelines such as *Mindset* where the majority of existing recommendations are constraining negatively-worded ‘do not’s’.

Second, the results suggest the need for strong and pro-active outreach work with U.S. journalists to encourage them to better follow core suicide reporting recommendations. Results from the present study, as well as related evidence, suggests that in particular, U.S. journalists should be encouraged to (i) not go into detail about method used; (ii) avoid romanticizing the act and (iii) include helpline numbers, on-line resources and further information about where to seek help. Journalists should also be encouraged to continue the observed trend to use suicide reporting as an opportunity to open dialogue and discussion about wider issues of suicide prevention and mental health, which can raise awareness and foster help-seeking [38].

Such action could be inspired by ongoing approaches employed in Canada. In collaboration with numerous organizations, the Mental Health Commission of Canada has created and implemented a number of programs to improve media reporting of mental illness and suicide. These include (i) the creation and distribution of *Mindset* to over 5 000 newsrooms and journalists; (ii) the creation of a freely-available on-line course on best practice mental health and suicide reporting for current journalists and journalism students; and (iii) regular visits to major journalism schools to conduct panel-based interactive educational sessions aimed at students and faculty. Recent research suggests that these activities may have been successful in significantly improving Canadian media coverage of mental illness and suicide in recent years [13, 25, 39, 40].

There are some limitations to this study. First, the study only included ten newspapers, a small proportion of the newspapers published in the United States. Articles in other newspapers or focused on a specific region may have given different results. That said, we included best-selling national and regional newspapers, which at least give an indication of general patterns in the U.S. suicide reporting landscape. Moreover, the number of newspapers in the present study is similar to that seen in the analogous studies from other jurisdictions mentioned in the introduction [13,17,18,19,20].

Second, we assessed U.S. articles’ adherence to recommendations created and published in Canada (*Mindset*). This was done to provide a valid and direct comparison between the two jurisdictions, and the *Mindset* recommendations do overlap considerably with other suicide reporting guidelines such as those produced by the World Health Organization. That said, *Mindset* has been widely distributed to Canadian journalists, and not to U.S. equivalents, perhaps explaining greater adherence in Canada.

Third, a single coder (the first author) was involved in the granular-level analysis of all the data. This involved the exercise of subjective judgment to evaluate the presence or absence of themes, introducing the possibility of observer bias. However we took many steps to reduce such bias. Firstly, the first author is a highly-trained analyst with considerable experience analyzing media coverage of suicide and mental illness [25,26], who was closely supervised by the second author who helped resolve ambiguities during the various stages of data analysis. Secondly, there was substantial agreement when a sub-sample of articles were double coded by the two authors following training. Thirdly, the coding and judgment calls were not complex in nature, involving simple yes/no responses to the presence or absence of simple words or themes; such non-complex judgements do not require complex coding procedures [41]. Moreover, using a single well-trained and well-supervised coder in such studies is often considered an advantage as it is cost-effective and reduces inter-rater variability, as long as this is accompanied by a check on reliability from a second coder, which was the case in the present study [32]. Likewise, this approach is commonly-used when analyzing media stories about suicide and mental illness [13,24,25]. Still, there may have been some minor observer bias in the study.

Finally, we focused on a small selection of newspapers within the mainstream media. This focus on one segment of the media (mainstream newspapers) is limiting given the diversity of media in the modern world. Reports in other forms of mainstream media such as television or radio may have given different results. Additionally, analysis of social media is vitally important to better understand suicide contagion [42] but was not conducted in the present study.

Indeed, there is a dire need for further research on the role of social media in suicide contagion as there has been a massive growth in on-line citizen journalism, user-generated content and widely shared social media commentaries on current events [43]. As such, journalists are no longer the sole gatekeepers to knowledge and information about suicide. Improving professional journalists’ reporting of suicide remains critical, however this is one part of an increasingly complex jigsaw. There is a need for further research to understand how to positively affect citizen ‘reporting’ and social media sharing about suicide.

## Conclusions

This study suggests that U.S. newspapers moderately adhered to best practice recommendations when reporting Robin Williams’ suicide. Many recommendations were consistently followed, however others were frequently flouted, including core recommendations not to go into details about the methods used and not to romanticize the act. The patterns of reporting in the U.S. media differed to patterns of reporting in the Canadian media, with Canadian newspapers having much higher adherence to suicide reporting guidelines. This pattern of reporting in the U.S. media may have contributed to suicide contagion in the U.S. in the months following Williams’ suicide. As such, new interventions targeted at the U.S. media and journalists may be necessary to improve suicide reporting, which may in turn reduce suicide contagion.

## Supporting information

S1 File Formalized codebook used to operationalize the mindset guidelines. (DOCX)

S1 Appendix List of newspapers included in the sample. (DOCX)

S2 Appendix List of newspaper articles included in sample. (DOCX)

S1 Data Excel spreadsheet. (XLSX)
