# Supplementary material for: Media coverage of Robin Williams’ suicide in the United States: A contributor to contagion?
Source: PLoS One. 2019 May 9;14(5):e0216543. doi: 10.1371/journal.pone.0216543 (PMC6508639; doi:10.1371/journal.pone.0216543)
Supplement: S1 File — (DOCX) [file pone.0216543.s001.docx]

| **Recommendation** | **Operationalization** |
| --- | --- |
| Do consider whether this particular death is newsworthy. | n/a |
| Do look for links to broader social issues. | The article includes discussion of social factors related to suicide such as isolation. |
| Do respect the privacy and grief of family or other survivors. | The article does not include comments from Williams’ family or friends. |
| Do include reference to their suffering. | The article describes any aspect of Williams’ past suffering prior to suicide. |
| Do tell others considering suicide how they can get help. | The article includes information about suicide resources, such as websites or helplines. |
| Do not shy away from writing about suicide. | n/a |
| Do not romanticize the act. | The article discusses suicide using factual language rather than in a glamourized/ sensationalized manner. |
| Do not jump to conclusions. | The article does not report that one specific factor ‘caused’ Williams’ suicide. |
| Do not suggest nothing can be done. | The article does not suggest the inevitability of suicide given Williams’ circumstances. |
| Do not go into details about the method used. | The article does not mention the method used in Williams’ suicide. |
| Do use plain words. | The article describes the suicide in simple language such as ‘died by suicide’. |
| Do not say the person committed suicide. | The article does not use the word ‘commit’ when reporting the suicide. |
| Do not call suicide successful or attempted suicide unsuccessful. | The article does not use the words ‘successful/ unsuccessful’ in relation to Williams’ suicide. |
| Do not use or repeat pejorative phrases. | The article does not express contempt through language like ‘the coward’s way out’. |

**S1. Formalized Codebook used to Operationalize the Mindset Guidelines**
