# Supplementary material for: Media coverage of Robin Williams’ suicide in the United States: A contributor to contagion?
Source: PLoS One. 2019 May 9;14(5):e0216543. doi: 10.1371/journal.pone.0216543 (PMC6508639; doi:10.1371/journal.pone.0216543)
Supplement: S1 Appendix — (DOCX) [file pone.0216543.s002.docx]

**Appendix 1: List of newspapers included in the sample**

1. Atlanta Journal-Constitution
2. Boston Globe
3. Independent Weekly
4. Los Angeles Times
5. Miami Herald
6. New York Times
7. Philadelphia Inquirer
8. USA Today
9. Washington Post
10. Washington Times
