# Supplementary material for: Media coverage of Robin Williams’ suicide in the United States: A contributor to contagion?
Source: PLoS One. 2019 May 9;14(5):e0216543. doi: 10.1371/journal.pone.0216543 (PMC6508639; doi:10.1371/journal.pone.0216543)
Supplement: S2 Appendix — (DOCX) [file pone.0216543.s003.docx]

**Appendix 2: List of newspaper articles included in sample**

| **A1** | *USA Today*, “Robin Williams bio coming from Henry Holt and Dave Itzkoff”, 3 September 2014. |
| --- | --- |
| **A2** | *USA Today*, “Psychologist helps comedians stand up to pressures, especially in wake of Williams' death”, 28 August 2014. |
| **A3** | *Los Angeles Times*, “Emmys 2014: Billy Crystal recalls departed friend Robin Williams”, 28 August 2014. |
| **A4** | *Philadelphia Inquirer*, “Check up: Penn doctor’s study links Parkinson’s and depression”, 24 August 2014. |
| **A5** | *Atlanta Journal-Constitution*, “Robin Williams’ death has us reflecting on our lives”, 23 August 2014. |
| **A6** | *USA Today*, “Robin Williams was cremated, his ashes scattered in San Francisco Bay”, 21 August 2014. |
| **A7** | *Miami Herald*, “Media should not glamorize suicide”, 18 August 2014. |
| **A8** | *Boston Globe*, “Parity in mental health care a must”, 17 August 2014. |
| **A9** | *Miami Herald*, “Robin Williams’ 10 best movies”, 16 August 2014. |
| **A10** | *Washington Post,* “The death of Robin Williams”, 16 August 2014. |
| **A11** | *Los Angeles Times*, “Los Angeles Times Sandy Banks column”, 16 August 2014. |
| **A12** | *Boston Globe*, “Robin Williams was in the early stages of Parkinson’s disease, his wife reports”, 15 August 2014. |
| **A13** | *Los Angeles Times*, “Robin Williams’ widow says actor was sober, had Parkinson’s at death”, 15 August 2014. |
| **A14** | *New York Times*, “Robin Williams is said to have had Parkinson’s”, 15 August 2014. |
| **A15** | *New York Times*, “The vulnerable showman”, 15 August 2014. |
| **A16** | *USA Today*, “Robin Williams was battling Parkinson’s, widow says”, 15 August 2014. |
| **A17** | *Los Angeles Times*, “Opinion: The details of Robin Williams’ death should not be covered up”, 14 August 2014. |
| **A18** | USA Today, “Robin Williams was in early stages of Parkinson’s disease, wife says”, 14 August 2014. |
| **A19** | USA Today, “Robin Williams: A link between genius, mental illness?”, 14 August 2014. |
| **A20** | *Miami Herald*, Michael Douglas' ex throws shade at Catherine Zeta Jones; wife says Robin Williams had Parkinson's; Chris Brown progress report”, 14 August 2014. |
| **A21** | *Miami Herald*, “Robin Williams’ choice”, 14 August 2014. |
| **A22** | *USA Today*, “Calls to crisis hotlines surge after Williams’ suicide”, 14 August 2014. |
| **A23** | *Los Angeles Times*, “Depression itself can be a symptom of Parkinson’s, experts say”, 14 August 2014. |
| **A24** | *Los Angeles Times*, “Officials defend release of graphic Robin Williams suicide details”, 14 August 2014. |
| **A25** | *USA Today*, “Robin Williams was sober, in early stages of Parkinson’s disease, wife says”, 14 August 2014. |
| **A26** | *Atlanta Journal-Constitution*, “YOUR HEALTH: Williams' death puts spotlight on suicide: Many people don't recognize the warning signs of depression”, 14 August 2014. |
| **A27** | *Los Angeles Times*, “Robin Williams’ image won’t fade from multiplexes soon”, 14 August 2014 |
| **A28** | *Washington Post*, “Older white men face higher suicide risk”, 13 August 2014. |
| **A29** | *Boston Globe*, “Comic mania can mask darkness with joy”, 13 August 2014. |
| **A30** | *Los Angeles Times*, “Robin Williams’ friends saw he was succumbing to depression”, 13 August 2014. |
| **A31** | *Washington Post*, “Coroner releases details in death of Robin Williams”, 13 August 2014. |
| **A32** | *Washington Post*, “Weighing in on celebrity deaths”, 13 August 2014. |
| **A33** | *USA Today*, “Robin Williams found hanged with a belt, cops say”, 13 August 2014. |
| **A34** | *Los Angeles Times*, “Robin Williams memorial grows outside ‘Mrs. Doubtfire’ house”, 13 August 2014. |
| **A35** | *New York Times*, “Robin Williams died by hanging, official says”, 13 August 2014. |
| **A36** | *New York Times*, “Busy working, Robin Williams fought demons”, 13 August 2014. |
| **A37** | *USA Today*, “Advocates hope Robin Williams’ death will spur discussion”, 13 August 2014. |
| **A38** | *Los Angeles Times*, “Robin Williams’ death: Coroner’s investigation will take weeks”, 13 Aug 2014. |
| **A39** | *Boston Globe*, “Williams death brings rush of emotions in city”, 13 August 2014. |
| **A40** | *USA Today*, “Nation and world news briefs”, 13 August 2014. |
| **A41** | *Washington Post*, “Actor Robin Williams - who, experts said, fit the profile of someone at high risk of committing suicide - killed himself by hanging, authorities said”, 13 August 2014. |
| **A42** | *USA Today*, “Oscar winner Robin Williams dies at 63”, 12 Aug 2014. |
| **A43** | *Los Angeles Times*, “Robin Williams dies in apparent suicide; actor, comic was 63”, 12 August 2014. |
| **A44** | *Los Angeles Times*, “Details of Robin Williams’ death generate backlash on social media”, 12 Aug 2014. |
| **A45** | *Los Angeles Times*, “Robin Williams: How to recognize suicide signs, where to get help”, 12 Aug 2014. |
| **A46** | *Los Angeles Times*, “Robin Williams hanged himself in bedroom with a belt, sheriff says”, 12 Aug 2014. |
| **A47** | *Boston Globe*, “A somber end for comic master Robin Williams”, 12 Aug 2014. |
| **A48** | *Washington Post*, “A satirist, a mimic, a dramatist”, 12 Aug 2014. |
| **A49** | *USA Today*, “Suicide a risk even for beloved characters like Williams”, 12 Aug 2014. |
| **A50** | *USA Today*, “Robin Williams’ death shocks Marin County neighbors”, 12 Aug 2014. |
| **A51** | *USA Today*, “Celebrating Robin Williams’ great life and career”, 12 Aug 2014. |
| **A52** | *Los Angeles Times*, “Robin Williams: Comedy Store, Laugh Factory, Improv pay tribute”, 12 Aug 2014. |
| **A53** | *Los Angeles Times*, “Fans mourn death of Robin Williams on Hollywood Walk of Fame”, 12 Aug 2014. |
| **A54** | *Philadelphia Inquirer*, “Robin Williams, 63, comic genius”, 12 Aug 2014 |
| **A55** | *Los Angeles Times*, “Robin Williams dies at 63; Oscar-winning actor, comic genius”, 12 Aug 2014. |
| **A56** | *USA Today*, “Robin Williams’ life was punctuated by struggles with alcohol, cocaine”, 12 Aug 2014. |
| **A57** | *Los Angeles Times*, “Robin Williams’ body was found by personal assistant, official says”, 12 Aug 2014. |
| **A58** | *Los Angeles Times*, “Robin Williams death: Actor last seen alive late Sunday, officials say”, 12 Aug 2014. |
| **A59** | *Miami Herald*, “Robin Williams’ death focuses attention on depression”, 12 Aug 2014. |
| **A60** | *Boston Globe*, “Robin Williams, frenetic comic with dramatic bona fides”, 12 Aug 2014. |
| **A61** | *USA Today*, “Robin Williams dead at 63 from apparent suicide”, 12 Aug 2014. |
| **A62** | *USA Today*, “Robin Williams: Six new movies he left behind”, 12 Aug 2014. |
| **A63** | *USA Today*, “Robin Williams co-stars saw loving side till the end”, 12 Aug 2014. |
